# Fluid-structure interaction analysis for abdominal aortic aneurysms: the role of multi-layered tissue architecture and intraluminal thrombus

**DOI:** 10.3389/fbioe.2025.1519608

**Published:** 2025-02-11

**Authors:** Xinhai Yue, Jiayi Huang, Ju Liu

**Affiliations:** Department of Mechanics and Aerospace Engineering, Southern University of Science and Technology, Shenzhen, Guangdong, China

**Keywords:** fluid-structure interaction, abdominal aortic aneurysm, intraluminal thrombus, multi-layered anisotropic tissue model, patient-specific modeling

## Abstract

**Introduction:**

Abdominal aortic aneurysm (AAA) is a life-threatening disease marked by localized dilatations of the infrarenal aortic wall. While clinical guidelines often use the aneurysm diameter as an indicator for surgical intervention, this metric alone may not reliably predict rupture risks, underscoring the need for detailed biomechanical analyses to improve risk assessments.

**Methods:**

We investigate the effects of the multi-layered tissue architecture and the intraluminal thrombus (ILT) on the wall stress distribution of AAA. Using fluid-structure interaction, we analyze the biomechanical responses of fusiform and saccular AAAs under three conditions: without ILT, with ILT but no tissue degradation, and with both ILT and tissue degradation.

**Results:**

The findings show that the media is the primary load-bearing layer, and the multi-layered model yields a more accurate stress profile than the single-layered tissue model. The ILT substantially reduces overall stress levels in the covered tissue, although its impact on the location of peak stress varies across different scenarios. Media degradation increases the stress in the intima and adventitia, but the cushioning effect of ILT largely mitigates this impact.

**Discussion:**

The results underscore the importance of incorporating the multi-layered tissue architecture and ILT in patient-specific analyses of AAA. These factors may improve the predictive capabilities of biomechanical assessments for rupture risk.

## 1 Introduction

Abdominal aortic aneurysms (AAAs) are focal dilatations of the infrarenal aortic wall, often caused by localized weakening of the vessel wall. While most AAAs are asymptomatic, progressive expansion can lead to rupture with severe, life-threatening medical consequences. Untreated, a ruptured aneurysm carries a mortality rate as high as 85%, and even with timely medical intervention, the mortality rate exceeds 30% (Kent, 2014). This underscores the critical importance of accurately assessing AAA rupture risk. Clinically, the maximum diameter is used as an indicator for rupture potential and surgical intervention. This criterion is known to underestimate the rupture risk of small AAAs, especially in female patients (Moll et al., 2011). Additionally, for some patients with large AAAs, the risks associated with surgery may outweigh the risk of aneurysm rupture (Lederle et al., 2002). Consequently, a comprehensive risk assessment of AAAs based on multiple factors is essential for optimized patient management.

The arterial wall consists of three distinct layers: the intima, media, and adventitia, each playing a critical role in maintaining vascular function and regulating hemodynamic forces (Holzapfel et al., 2000). The *intima*, composed primarily of endothelial cells, provides a smooth surface for blood flow; the *media*, rich in smooth muscle cells, elastin, and collagen, modulates vascular diameter and elasticity, enabling the vessel to endure arterial pressure; the *adventitia*, composed of loose connective tissue, provides tensile strength and flexibility, thereby maintaining the structural integrity of vessels. Vascular smooth muscle cells within the media play a crucial role in processing the extracellular matrix, including the formation of elastin and collagen fibers. The degradation of these fibers and the reduction of the smooth muscle cells significantly contribute to the progression of AAAs.

To describe the material properties of arterial walls, Holzapfel et al. proposed using an isotropic model to represent the non-fibrous matrix and employed an exponential function to describe the fiber behavior (Holzapfel et al., 2000). Building on that, Gasser et al. introduced a fiber dispersion factor, capturing the symmetric fiber distribution around a mean orientation (Gasser et al., 2006). Holzapfel et al. later refined this model by incorporating a non-symmetric orientation density function, offering a more detailed representation of fiber distributions (Holzapfel et al., 2015). Several experimental studies have applied these models, or their variants, to determine layer-specific material parameters for both healthy and aneurysmal vessel walls (Weisbecker et al., 2012; Sassani et al., 2015; Niestrawska et al., 2016).

Intraluminal thrombus (ILT) refers to blood clots formed within vessels. Approximately 75% of AAAs contain thrombi (Tong and Holzapfel, 2015). While ILT typically functions to stop bleeding and repair blood vessels as part of the normal physiological process, it complicates the rupture risk assessment. Some studies suggest that aneurysm rupture may be correlated with increased ILT volume (Hans et al., 2005; Haller et al., 2018). A thicker ILT may lead to hypoxia in the adjacent AAA tissue, which may result in the degradation of the extracellular matrix and a significant reduction of wall strength (Vorp et al., 2001). Additionally, ILT may promote vascular smooth muscle cell apoptosis, leading to a thinner vascular wall beneath it (Koole et al., 2013). As a result, ILT can contribute significantly to AAA growth and elevate the rupture risk. Conversely, from a biomechanical standpoint, the thrombi also reduce the stress on the underlying AAA tissue, which may lower the rupture risk (Wang et al., 2002; Di Martino and Vorp, 2003; Throop et al., 2022). As a result, when assessing the rupture risk of AAA, the impact of ILT needs to be thoroughly studied.

Finite element analysis and fluid-structure interaction (FSI) are valuable tools for assessing the rupture risks of AAAs and understanding the biomechanical impact of ILT by evaluating the wall stress of AAAs. Traditional finite element analysis employs an idealized geometry and applies uniform internal pressure to simulate the load exerted by blood flows. Although this method offers computational simplicity, it fails to account for the pulsatile nature of blood flow, a critical factor in both AAA rupture and ILT formation. Consequently, this simplification may lead to an incomplete assessment of rupture risk. FSI offers more realistic simulation results by integrating the interaction between fluid (e.g., blood) and structure (e.g., arterial wall and ILT). It is noted that the enhanced accuracy of FSI comes at the cost of increased modeling complexities. Early studies relied on idealized geometric models and isotropic material assumptions for both the aneurysm and ILT (Di Martino and Vorp, 2003; Scotti et al., 2005). Although these models provided valuable insights, they lacked the fidelity to accurately represent the patient-specific geometries and physiologically realistic material properties.

With advancements in medical imaging, patient-specific geometries of AAAs and ILTs have been incorporated into both finite element and FSI analyses, enabling more accurate predictions of wall stress distribution (Maier et al., 2010). This enhanced modeling capability is crucial for assessing rupture risk, particularly when combined with the spatial distribution of wall strength. Given the anisotropic nature of vascular tissues, contemporary analyses have increasingly adopted anisotropic material models (Rodríguez et al., 2009; Riveros et al., 2015; Rissland et al., 2009; Xenos et al., 2010; Xenos et al., 2015). Studies have demonstrated that stress magnitudes derived from anisotropic models are significantly higher than those from isotropic models, underscoring the importance of incorporating anisotropic models to accurately represent material properties (Rodríguez et al., 2009; Rissland et al., 2009). Moreover, accounting for the layered tissue structure allows for a more refined transmural stress distribution, as each layer exhibits distinct mechanical behaviors essential for a comprehensive understanding of AAA biomechanics (Simsek and Kwon, 2015; de Lucio et al., 2021). Notably, degeneration of the media has been incorporated to explore mechanisms underlying AAA initiation and progression (Simsek and Kwon, 2015). Regarding the impact of ILT on AAA stress conditions, most studies indicated that ILT significantly affects the stress distribution on the aneurysm wall, with the maximum wall stress typically occurring in regions where the ILT is thinnest (Riveros et al., 2015). Interestingly, one study noted that while ILT affects stress magnitude, it does not significantly alter the site of the peak stress (Xenos et al., 2015).

Given these inconsistent findings, it is evident that current models may not fully capture the complexities of AAA biomechanics. Our research aims to address these gaps by developing a more comprehensive approach that incorporates the layered structure of the vascular wall, anisotropic material properties, ILT, and ILT-induced media degradation. To achieve these objectives, this work is organized as follows. Section 2.1 presents the geometric modeling method for ILT and the layered architecture of the AAA wall. It also details the procedure for generating a local coordinate system for the solid mesh, which facilitates the description of the anisotropic tissue models. Section 2.2 presents the FSI formulations employed in this study, including the physiological boundary conditions for the simulations. Section 2.3 introduces the material models applied in the simulations, including the anisotropic hyperelastic model for the AAA tissue and the isotropic hyperelastic model for the ILT. Section 3 presents the FSI analyses conducted on both fusiform and saccular AAAs. For cases with multi-layered tissue model, layer-specific material parameters are used to describe the intima, media, and adventitia of the AAA wall. We compare the maximum principal stress (MPS) distribution, focusing on layer-specific stress distributions under three conditions: (1) without ILT, (2) with ILT but no tissue degradation, and (3) with both ILT and degradation. We discuss our findings in Section 4 and draw conclusions in Section 5. Limitations of this study is discussed in Section 6.

## 2 Methods

### 2.1 Geometric modeling

We start by presenting an image-based geometric modeling pipeline for AAA with ILT. The geometric model is essential for the subsequent FSI analysis. The process begins by identifying the pathline for the lumen of interest from medical images. Along the pathline, two-dimensional segmentations are employed on the planes perpendicular to the pathline to extract the contour lines for the lumen. The contour lines are represented by closed B-spline curves, defined by a series of control points. Lofting these contour lines along the pathline results in a smooth tubular spline surface. It describes the luminal surface and is also regarded as the interior tissue wall surface without the presence of ILT. To construct the exterior tissue wall surface, a new set of contour control points is generated. These points are collinear with the lumen centroid and the interior surface contour control points, with their distance to the centroid increased by a thickness value δ . Lofting these newly generated contour lines generates the exterior tissue wall surface. Planar surfaces at the inlet and outlet are generated to close the volume and complete the boundary representations (B-Reps) for the lumen and tissue. For multi-branched vessels, the same procedures are repeated for each vessel, and boolean addition operations are performed to combine the resulting surfaces. Readers may refer to Sun et al. (2025a) for more technical details on the procedures described above.

**FIGURE 1:**
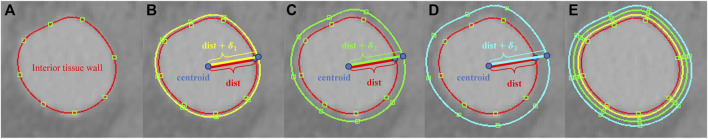
**(A)** A contour line for the lumen surface on a 2D slide of a medical image; **(B)** the contour line for the exterior surface of the intima generated by scaling with the thickness $\delta_{1}$ ; **(C)** the contour line for the exterior surface of the media generated by scaling with the thickness $\delta_{2}$ ; **(D)** the contour line for the exterior surface of the adventitia generated by scaling with the thickness $\delta_{3}$ ; **(E)** the resulting contour lines for the multi-layered vascular tissue.

To account for the multi-layered architecture of vascular tissues, we extend the above procedure to construct additional contour lines that delineate the intima, media, and adventitia. As shown in Figure 1, starting from the contour lines of the lumen surface, three new sets of contour lines are generated using the thickness $\delta_{1}$ , $\delta_{2}$ , and $\delta_{3}$ . They are used for generating the exterior wall surfaces of the intima, media, and adventitia. The exterior wall surface of the inner layer also serves as the interior wall surface of the adjacent outer layer. Finally, by closing the planar surfaces at the inlet and outlet, we obtain the B-Reps for the tissue layers and the lumen.

**FIGURE 2:**
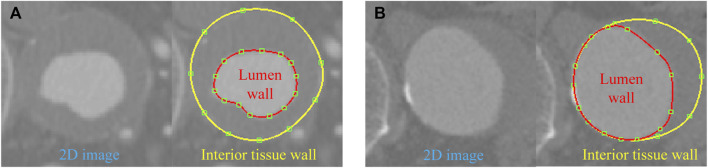
Contour lines delineated for the lumen and interior tissue wall surfaces in two cases: **(A)** ILT completely covers the aneurysm wall; **(B)** ILT covers a portion of the aneurysm wall.

When the vascular vessel contains ILT, the lumen wall surface differs from the interior tissue wall surface, necessitating a refinement of the above procedures. On planes perpendicular to the pathline, we extract the contour lines for both the lumen wall and the interior wall surface of the tissue (Figure 2). These contours are lofted to generate the lumen surface and interior tissue surface, respectively. With the inlet and outlet surfaces closed, we obtain two distinct tubular volumes based on the two wall surfaces. A boolean subtraction operation is then performed to isolate the ILT volume, preparing it for mesh generation.

With the geometric representation established, a mesh generation algorithm can be applied for discretization. We start by generating the mesh for the luminal domain, and a boundary layer mesh may be created using the advancing layer method. Once the lumen mesh is obtained, we proceed to create the mesh for the ILT, if present; otherwise we generate the mesh for the intima. At this stage, the discrete B-Rep is strictly adhered to, meaning that the luminal mesh remains unmodified. In doing so, the meshes of both domains match across their interface. In FSI analysis, the matching interface property conveniently ensures the proper coupling conditions (Sun et al., 2025b). Analogously, with the mesh of the intima, one may proceed to generate the mesh for the media which conforms to the intima mesh. Finally, the mesh for the adventitia can be generated with a conforming discrete representation of the interface between adventitia and media.

**FIGURE 3:**
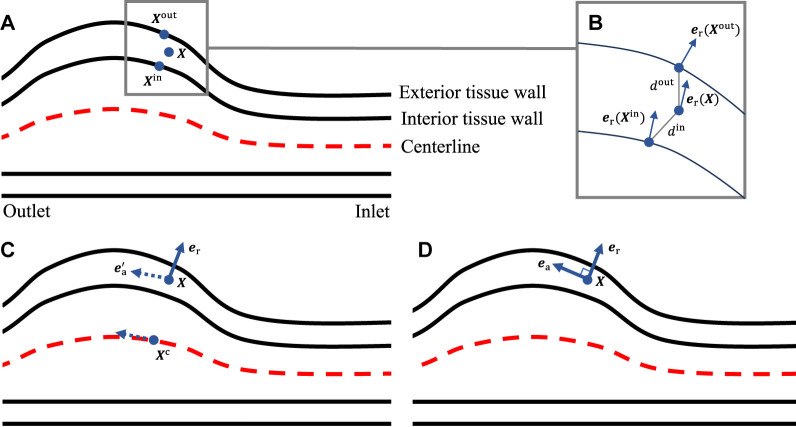
**(A)** Locating the closest points on the interior and exterior tissue wall; **(B)** evaluating the local radial direction; **(C)** identifying the closest point on the centerline to get the pseudo-local axial direction; **(D)** evaluating the local axial direction.

To characterize the anisotropic behavior of the tissue, we define the local radial, circumferential, and axial directions using a morphology-based approach introduced in Sun et al. (2025a). Here we briefly outline the procedures for a single layer, and one needs to repeat the procedures for each layer. We first compute the outward normal vectors for the mesh nodes on both the interior and exterior wall surfaces of the tissue layer. This can be achieved using mature algorithms developed for polygon surfaces. For a generic point X located within the tissue, we identify the closest mesh nodes $X^{\text{in}}$ on the interior wall surface and $X^{\text{out}}$ on the exterior wall surface (Figure 3A). The distances between X and the two mesh nodes are denoted as $d^{\text{in}}$ and $d^{\text{out}}$ , respectively. The outward normal vectors at the two mesh nodes are denoted as $e_{\text{r}}\left(X^{\text{in}}\right)$ and $e_{\text{r}}\left(X^{\text{out}}\right)$ . As shown in Figure 3B, the outward normal vector at X , denoted as $e_{\text{r}}\left(X\right)$ , is a weighted average of $e_{\text{r}}\left(X^{\text{in}}\right)$ and $e_{\text{r}}\left(X^{\text{out}}\right)$ , that is,

$$e_{\text{r}}\left(X\right)≔\omega^{\text{in}}e_{\text{r}}\left(X^{\text{in}}\right)+\omega^{\text{out}}e_{\text{r}}\left(X^{\text{out}}\right),$$

where the weights $\omega^{\text{in}}$ and $\omega^{\text{out}}$ are defined as

$$\omega^{\text{in}}≔\frac{d^{\text{in}}}{d^{\text{in}}+d^{\text{out}}}\;\text{and}\;\omega^{\text{out}}≔\frac{d^{\text{out}}}{d^{\text{in}}+d^{\text{out}}},$$

respectively. Next, we extract the centerline of the lumen. For the point X , we identify its closest point $X^{\text{c}}$ on the centerline. The tangential vector $e_{\text{a}}^{\prime }$ at the point $X^{\text{c}}$ along the centerline, pointing towards the distal end, is determined (Figure 3C). Although the vector $e_{\text{a}}^{\prime }$ does not directly represent the axial direction at point X , it assists in defining the local circumferential direction $e_{\text{c}}$ by taking the cross product with $e_{\text{r}}$ . In the last, the local axial direction $e_{\text{a}}$ is calculated as $e_{\text{r}}\times e_{\text{c}}$ , as illustrated in Figure 3D.

**FIGURE 4:**
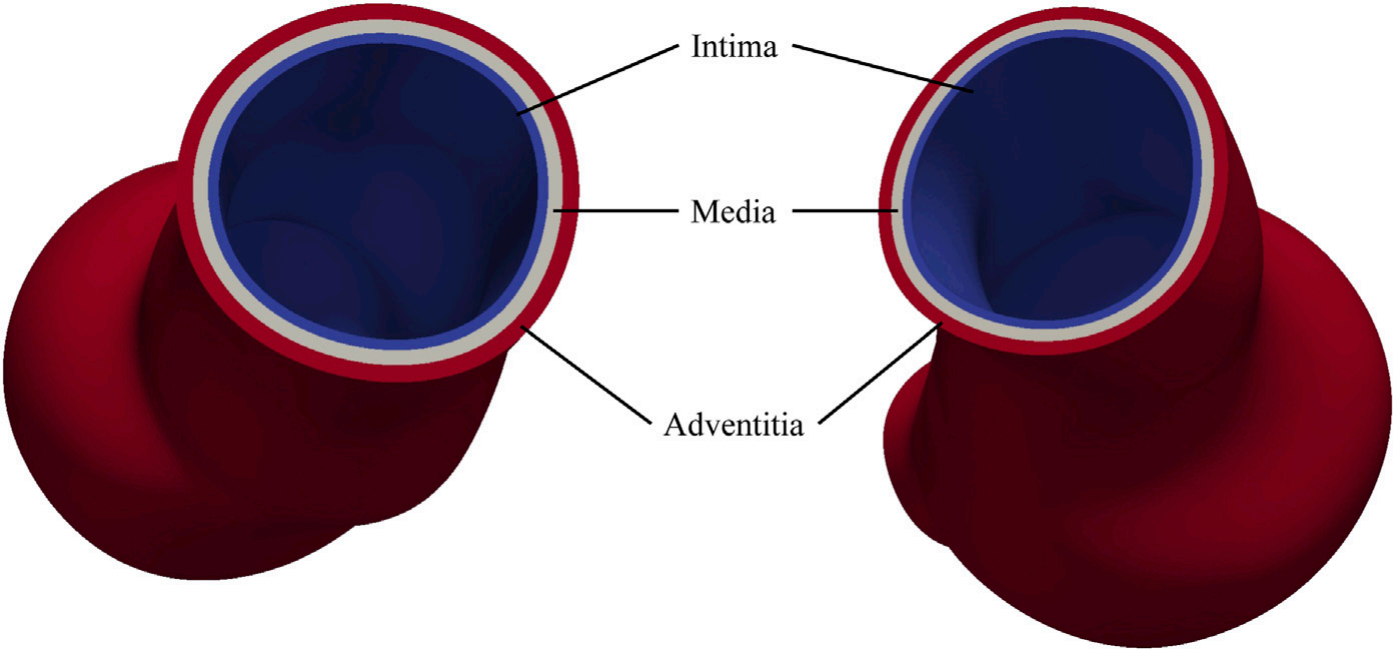
Multi-layered AAA geometric models.

**FIGURE 5:**
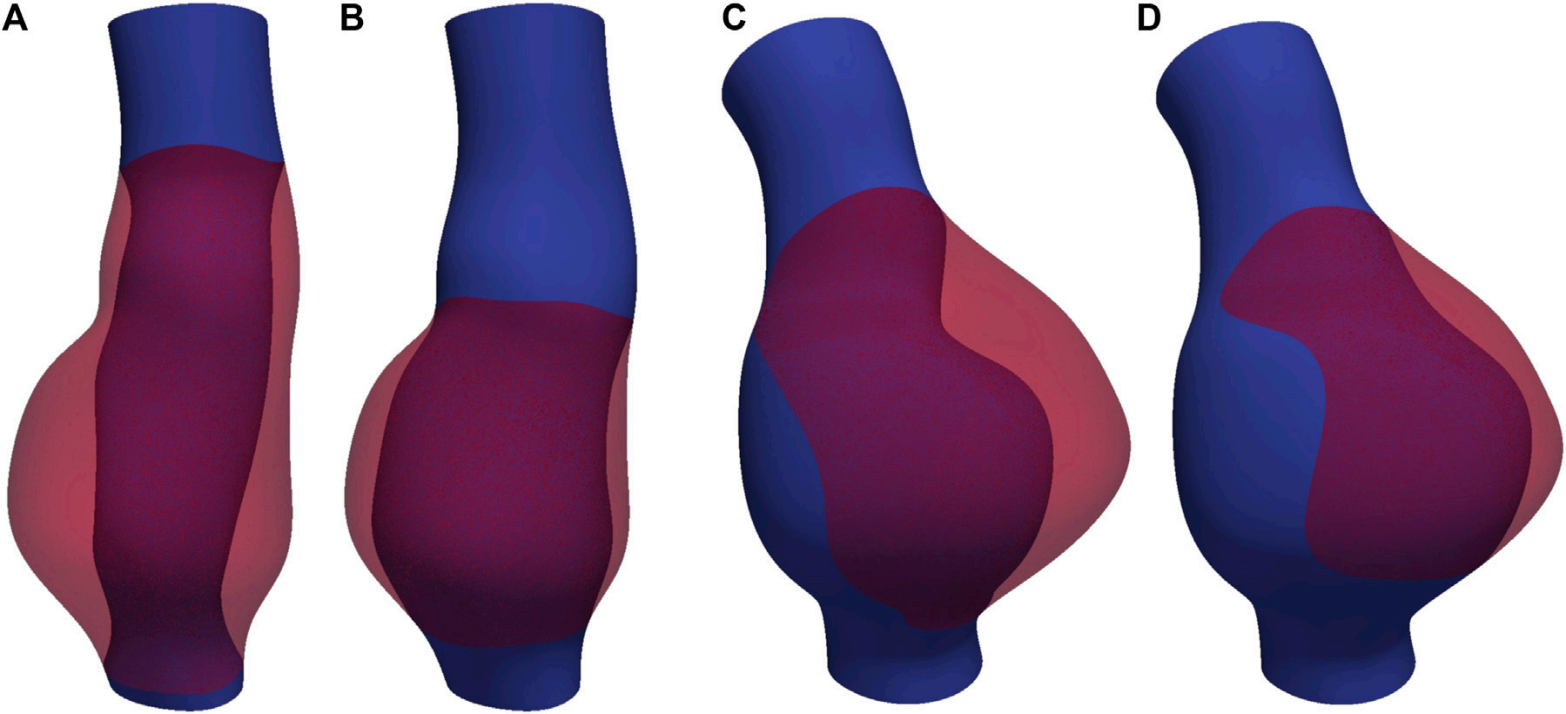
The lumen and ILT of **(A)** fAAA with the original volume of ILT, **(B)** fAAA with a smaller volume of ILT, **(C)** sAAA with the original volume of ILT, and **(D)** sAAA with a smaller volume of ILT.

**FIGURE 6:**
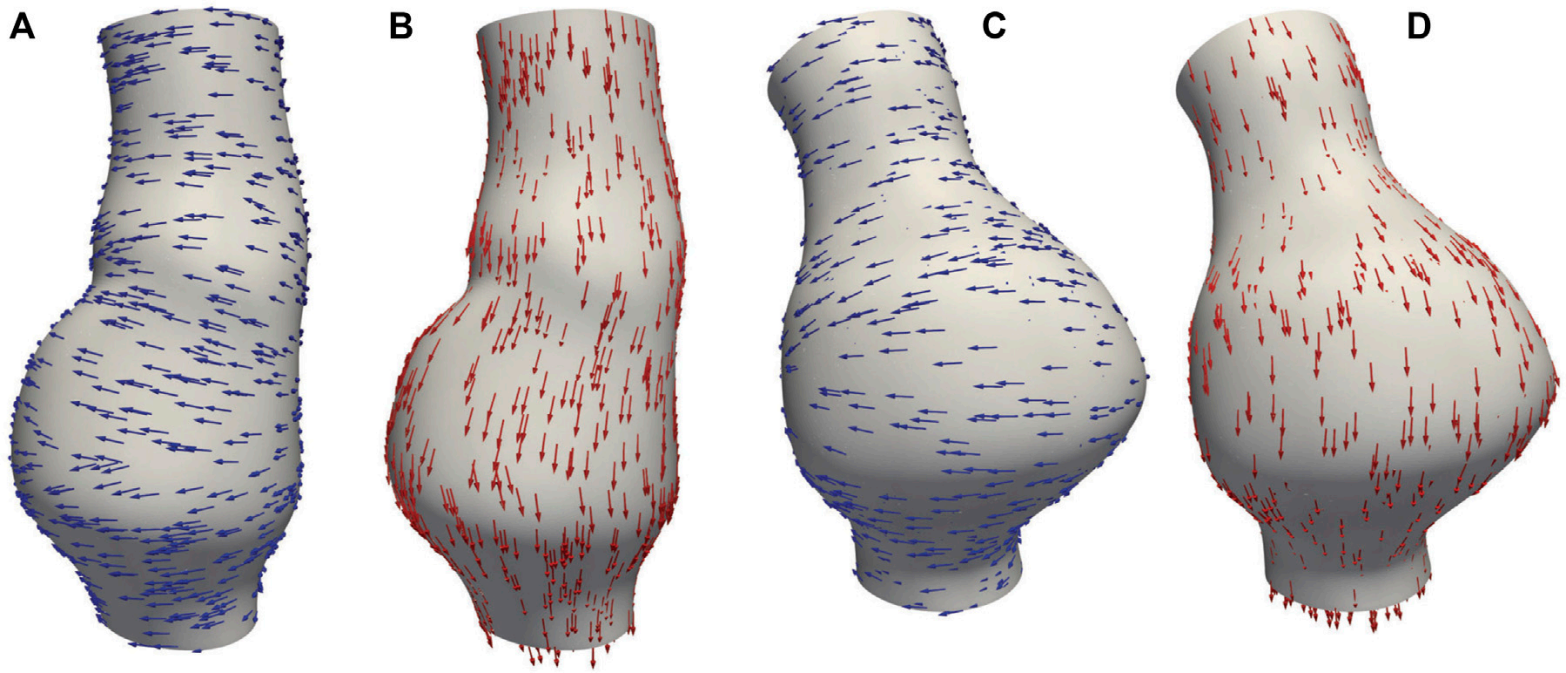
**(A)** Circumferential basis vectors of fAAA; **(B)** axial basis vectors of fAAA; **(C)** circumferential basis vectors of sAAA; **(D)** axial basis vectors of sAAA.

In this study, CT images from VMR (2024) are used to construct the geometries of two aneurysmal models: a fusiform (fAAA) and a saccular (sAAA) model^1^. The fAAA is taken from a 70-year-old male, with a maximum diameter of 3.65 cm. The sAAA is taken from a 48-year-old male, with a maximum diameter of 5.0 cm. Both contain ILT. It should be note that the CT images do not provide information on the tissue thickness. Therefore, the thickness values from the literature are adopted in this study. For geometries based on the single-layered tissue model, the tissue thickness is set to 2.69 mm. For the multi-layered tissue model, the intima, media, and adventitia thicknesses are set to 0.68 mm, 0.94 mm, and 1.07 mm, respectively (Weisbecker et al., 2012; de Lucio et al., 2021). The multi-layered geometric models are shown in Figure 4. For geometries with ILT, we first construct the original ILT volume using the aforesaid procedure. Two additional geometries, each with a smaller ILT volume, are created by virtually enlarging the lumen contours. The lumen and ILT geometries are shown in Figure 5. The mesh is generated using linear tetrahedral elements. After mesh generation, the local basis vectors are established, with the local circumferential and axial directions shown in Figure 6.

### 2.2 Formulation of the FSI problem

In this section, we start by introducing the governing equations for both the solid and fluid subproblems. The time interval of interest is (0,T) with the final time T>0 . Let $\Omega_{X}\subset R^{3}$ be the initial or referential configuration. Let $\Omega_{x}\subset R^{3}$ denote the current configurations, which is the image of $\Omega_{X}$ under the motion given by the mapping $x=\varphi \left(X,t\right)=\varphi_{t}\left(X\right)\in \Omega_{x}$ with $X\in \Omega_{X}$ . The displacement and velocity of the material particle initially located at X is defined as U≔x−X and V≔dU/dt , where we use d(⋅)/dt to represent the total time derivative. The deformation gradient is defined as $F≔\partial \varphi_{t}\left(X\right)/\partial X$ . The Jacobian determinant and the right Cauchy-Green deformation tensor are given by J≔det(F) and $C≔F^{T}F$ . Oftentimes, the deformation gradient is multiplicatively decomposed into the volumetric and isochoric parts, that is, $F=J^{1/3}\tilde{F}$ , with $\tilde{F}$ characterizing the volume-preserving deformation. Correspondingly, the unimodular right Cauchy-Green deformation tensor is defined as $\tilde{C}≔{\tilde{F}}^{T}\tilde{F}$ . We introduce P to represent the thermodynamic pressure on the initial configuration. The above kinematic and thermodynamic quantities are utilized in the formulation of the governing equations for both fluids and solids (Liu and Marsden, 2018; Sun et al., 2025b).

#### 2.2.1 Governing equations

The fluid subproblem characterizes the blood flow and is governed by the incompressible Navier-Stokes equations written in the arbitrary Lagrangian-Eulerian formulation (Liu and Marsden, 2018). Regarding the tissue and ILT, we treat them as elastic materials with distinct properties. We introduce a superscript i to indicate the quantities of the i -th solid sub-domain for 1≤i≤N , with N being the total number of solid sub-domains. When ILT is not present, the value of N equals the number of tissue layers; when the ILT is included, N equals the number of layers plus one. The solid sub-domains are numbered sequentially from the exterior to the interior. For instance, in a multi-layered tissue model with ILT, the adventitia, media, intima, and ILT are numbered as 1, 2, 3, and 4, respectively. The momentum and mass balance equations of the solid subproblems are stated as

$$\rho_{0}^{i}\frac{dV}{dt}-\nabla_{X}\cdot \left(FS^{i}\right)-\rho_{0}^{i}B=0,in\;\Omega_{X}^{i},$$ (1)

$$J\beta^{i}\left(P\right)\frac{dP}{dt}+\nabla_{X}:\left(JF^{-T}\right)=0,in\;\Omega_{X}^{i},$$ (2)

for i=1,…,N . In the above, $\rho_{0}^{i}$ is the density of the i -th material in the initial configuration, $\beta^{i}$ is the isothermal compressibility factor, and the second Piola-Kirchhoff stress $S^{i}$ is represented as $S^{i}=S_{\text{d}}^{i}-PJC^{-1}$ . The system (Equations 1, 2) is closed if the constitutive relations for the isothermal compressibility factor $\beta^{i}$ and the stress $S_{\text{d}}^{i}$ are provided. This is achieved through the specification of a thermodynamic potential $G^{i}\left(C,P\right)$ , known as the Gibbs free energy. It is often represented in the following additively split form

$$G^{i}\left(C,P\right)=G_{\text{d}}^{i}\left(C\right)+G_{\text{v}}^{i}\left(P\right),$$

and the constitutive relations for $\beta^{i}$ and $S_{\text{d}}^{i}$ can be given by

$$\beta^{i}=-\frac{\partial^{2}G_{\text{v}}^{i}}{\partial P^{2}}/\frac{\partial G_{\text{v}}^{i}}{\partial P}\;\text{and}\;S_{\text{d}}^{i}=2\frac{\partial G_{\text{d}}^{i}}{\partial C},$$

respectively. The forms of the free energies $G_{\text{d}}^{i}$ and $G_{\text{v}}^{i}$ completely characterize the material behavior and will be detailed in Section 2.3 for the vascular tissue and ILT. Interested readers may refer to (Liu and Marsden, 2018) for the background of the governing equations.

#### 2.2.2 Coupling conditions

The fluid-solid interface is the intersection between the fluid subdomain and the union of all solid subdomains. The j -th solid-solid interface is the intersection of $\Omega_{X}^{j}$ and $\Omega_{X}^{j+1}$ , where the index j ranges from 1 to N−1 . At the fluid-solid and solid-solid interfaces, the following conditions are imposed for proper coupling of different physical subproblems. First, the velocity v is demanded to be continuous across the interfaces, and this is often known as the *kinematic* coupling condition. This is conveniently achieved with the aid of the mesh compatibility, which ensures the nodes coincide on the interfaces between two physical sub-domains. The kinematic coupling condition is naturally satisfied by enforcing the velocity degrees-of-freedom on these nodes to be identical.

Second, the *dynamic* coupling condition demands the traction exerted by both domains on their interface must be equal in magnitude but opposite in direction. Again, with the mesh continuity, the continuity of the test functions across the interfaces leads to the satisfaction of the dynamic coupling condition in the variational sense. It needs to be pointed out that this condition results in a pressure jump across the interfaces. To properly account for the pressure discontinuity, a set of additional pressure nodes needs to be introduced over the interfaces (Sun et al., 2025b).

#### 2.2.3 Numerical settings

In the numerical treatment of the FSI problem, we employ equal-order interpolations for both the velocity and pressure. The variational multiscale formulation is invoked to provide the mechanisms of large eddy simulation and pressure stabilization (Liu and Marsden, 2018). Regarding the temporal discretization, the JWH-generalized- α method is adopted, as it is a robust option for multiphysics problems (Jansen et al., 2000; Sun et al., 2025b).

In the FSI analysis, the vessel wall on the inlet and outlet plane surfaces is fully clamped. The exterior wall surface of the vessel is set to be traction-free. A parabolic velocity profile is applied at the inlet with a pulsatile flow rate. On the outlet, a lumped parameter model is introduced to mimic the response of the downstream vasculature. In this work, we adopt the resistance model for the single-outlet problem, and it is given by p(t)=RQ(t)+P , where R is the resistance, Q(t) is the flow rate on the outlet surface, and P is the distal reference pressure.

### 2.3 Material models

In this study, both the ILT and AAA tissue are modeled as quasi-incompressible hyperelastic materials. We momentarily ignore the superscript i to simplify the discussion. The volumetric part of the energy $G_{\text{v}}$ for all solid materials takes the form

$$G_{\text{v}}\left(P\right)=\frac{-P^{2}+P\sqrt{P^{2}+K^{2}}}{2K}-\frac{K}{2}\ln\left(\frac{\sqrt{P^{2}+K^{2}}-P}{K}\right),$$ (3)

with K being the bulk modulus. It leads to the following constitutive relations for the isothermal compressibility factor,

$$\beta \left(P\right)={\left(P^{2}+K^{2}\right)}^{-\frac{1}{2}}.$$

It is worth mentioning that (Equation 3) is related to the volumetric energy $K\left(J^{2}-1-2\ln J\right)/4$ via a Legendre transformation (Liu and Marsden, 2018).

The ILT is modeled by an isotropic hyperelastic material (Di Martino and Vorp, 2003; Rodríguez et al., 2009) with the energy $G_{\text{d}}$ taking the form

$$G_{\text{d}}\left(C\right)=c_{1}\left({\tilde{I}}_{2}-3\right)+c_{2}{\left({\tilde{I}}_{2}-3\right)}^{2},$$

where ${\tilde{I}}_{2}≔\left[{\left(tr\tilde{C}\right)}^{2}-tr\left({\tilde{C}}^{2}\right)\right]/2$ is the second principal invariant of $\tilde{C}$ , and the parameters $c_{1}$ and $c_{2}$ are stress-like moduli. The three layers of the AAA tissue are modeled as a fiber-reinforced anisotropic hyperelastic material (Sun et al., 2025a), and the free energy $G_{\text{d}}$ is given by

$$G_{\text{d}}\left(C\right)=\frac{\mu }{2}\left({\tilde{I}}_{1}-3\right)+\underset{l=4,6}{\sum }\frac{k_{1}}{2k_{2}}\left[\exp\left\{k_{2}{\left[\kappa I_{1}+\left(1-3\kappa \right)I_{l}-1\right]}^{2}\right\}-1\right].$$ (4)

In this model, μ is a modulus with the unit in stress, governing the isotropic response of the non-fibrous matrix; the parameter $k_{1}$ has the unit of stress and scales the fiber contribution to the stiffness; the dimensionless parameter $k_{2}$ controls the exponential stiffness increase with the fiber stretch; the dimensionless parameter κ∈[0,1/3] characterizes the fiber dispersion. The associated invariants are defined as

$${\tilde{I}}_{1}≔tr\tilde{C},\;I_{1}≔trC,\;I_{4}≔C:\left(a_{0}⊗a_{0}\right),\;\text{and}\;I_{6}≔C:\left(b_{0}⊗b_{0}\right).$$

**FIGURE 7:**
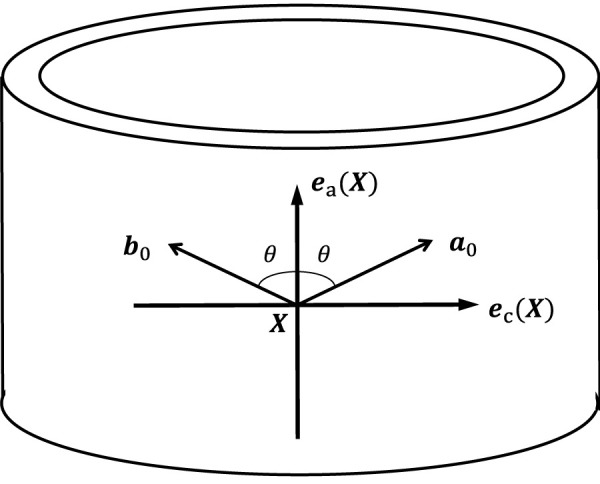
Illustration of the mean orientations of the two fiber families within the plane spanned by $e_{\text{a}}$ and $e_{\text{c}}$ .

The unit-length vectors $a_{0}$ and $b_{0}$ define the mean orientations of the two fiber families, respectively. We assume the fibers lie within the plane spanned by the local axial and circumferential directions and are symmetrically oriented with respect to the axial direction. Let θ denote the angle between the fiber mean orientation and local axial direction (see Figure 7). The vectors $a_{0}$ and $b_{0}$ can be expressed as

$$a_{0}\left(X\right)=\cos\theta e_{\text{a}}\left(X\right)+\sin\theta e_{\text{c}}\left(X\right),\;b_{0}\left(X\right)=\cos\theta e_{\text{a}}\left(X\right)-\sin\theta e_{\text{c}}\left(X\right).$$

Combined with the local basis vectors generated in Section 2.1, we may define the mean orientations of the two fiber families at each quadrature point for a patient-specific model.

## 3 Results

In this section, we perform FSI analysis and discuss the results. Section 3.1 compares the results obtained with single-layered versus multi-layered wall models; Section 3.2 examines the influence of ILT using multi-layered wall model by comparing cases with and without ILT; in Section 3.3, we investigate the media degradation caused by ILT by virtually introducing a degradation zone in the media. In all three sections, the average MPS is calculated to capture the overall stress distribution in the region of interest, enabling comparisons across cases. In Sections 3.1, 3.2, we also identify the location of the maximum MPS, which is essential for determining the regions within the highest rupture risk. Comparing these high-stress locations across cases, we gain insights into the influence of different factors on wall stress distribution.

In this study, the fluid density is set to $1.0\;g\cdot {cm}^{-3}$ and its dynamic viscosity is set to 0.04poise . The material parameters of the tissue and ILT are listed in Table 1. We mention that the material parameters of the ILT are adopted from the prior studies (Di Martino and Vorp, 2003), and the parameters of the tissue layers are given by layer-specific uniaxial tensile tests of AAA tissues (Sassani et al., 2015). The tests of (Sassani et al., 2015) were conducted on samples from patients undergoing open repair of AAAs. Each layer underwent uniaxial tensile tests in both axial and circumferential directions under physiological conditions. Material parameters were obtained using a nonlinear least-squares fitting procedure based on the material model presented in (Gasser et al., 2006). The fitting process incorporated both circumferential and axial data simultaneously to ensure consistency. The parameters for the single-layered wall model are obtained by averaging the parameters of the three layers, with weights based on their thicknesses. For the degraded media, the parameters μ and $k_{1}$ are set to 5% of their original values.

**TABLE 1:** The material parameters of ILT, intima, media, adventitia, and single-layered AAA wall.

	$\rho_{0}\left(g\cdot {cm}^{-3}\right)$	K(kPa)	$c_{1}\left(kPa\right)$	$c_{2}\left(kPa\right)$			
ILT	1.0	366.67	28.0	28.6			

	$\rho_{0}\left(g\cdot {cm}^{-3}\right)$	K(kPa)	μ(kPa)	$k_{1}\left(kPa\right)$	$k_{2}\left(-\right)$	κ(−)	θ(°)
Single-layered wall	1.0	646.80	39.6	1,804.20	129.52	0.26	57.5
Intima	1.0	542.27	33.2	2,845.95	204.42	0.30	49.2
Media	1.0	689.27	42.2	978.60	110.69	0.21	62.2
Adventitia	1.0	676.20	41.4	1,867.50	98.46	0.28	58.7
Degradation zone	1.0	34.46	2.11	48.93	110.69	0.21	62.2

To gain insights into the above material models and parameters, we consider a thin-walled tube problem as a preliminary analytical test of the anisotropic material model (Gasser et al., 2006). Assuming incompressibility, the internal pressure $p^{\text{in}}$ , circumferential stretch $\lambda_{\text{c}}$ , and axial stretch $\lambda_{\text{a}}$ are related by the following equations,

$$\lambda_{\text{a}}\frac{\partial {\hat{G}}_{\text{d}}}{\partial \lambda_{\text{a}}}-\frac{\lambda_{\text{a}}{\left(\lambda_{\text{c}}R-H/2\lambda_{\text{a}}\lambda_{\text{c}}\right)}^{2}}{2HR}p^{\text{in}}=0,$$ (5)

$$\lambda_{\text{c}}\frac{\partial {\hat{G}}_{\text{d}}}{\partial \lambda_{\text{c}}}-\left(\frac{\lambda_{\text{c}}^{2}\lambda_{\text{a}}R}{H}-\frac{1}{2}\right)p^{\text{in}}=0,$$ (6)

where R represents the mean radius of the tube, and H denotes the wall thickness. We set R=10.0 mm and H=0.9 mm to mimic a short segment of the infrarenal aorta. The modified free energy ${\hat{G}}_{\text{d}}$ is derived based on the model (Equation 4) and adopts the form,

$${\hat{G}}_{\text{d}}\left(\lambda_{\text{a}},\lambda_{\text{c}}\right)=\frac{\mu }{2}\left({\hat{I}}_{1}-3\right)+\frac{k_{1}}{k_{2}}\left[\exp\left\{k_{2}{\left[\kappa {\hat{I}}_{1}+\left(1-3\kappa \right){\hat{I}}_{4}-1\right]}^{2}\right\}-1\right],$$

where the invariants ${\hat{I}}_{1}$ and ${\hat{I}}_{4}$ are given as,

$${\hat{I}}_{1}=\lambda_{\text{a}}^{2}+\lambda_{\text{c}}^{2}+1/{\left(\lambda_{\text{a}}\lambda_{\text{c}}\right)}^{2},\;{\hat{I}}_{4}=\lambda_{\text{a}}^{2}{cos}^{2}\theta +\lambda_{\text{c}}^{2}{sin}^{2}\theta .$$

**FIGURE 8:**
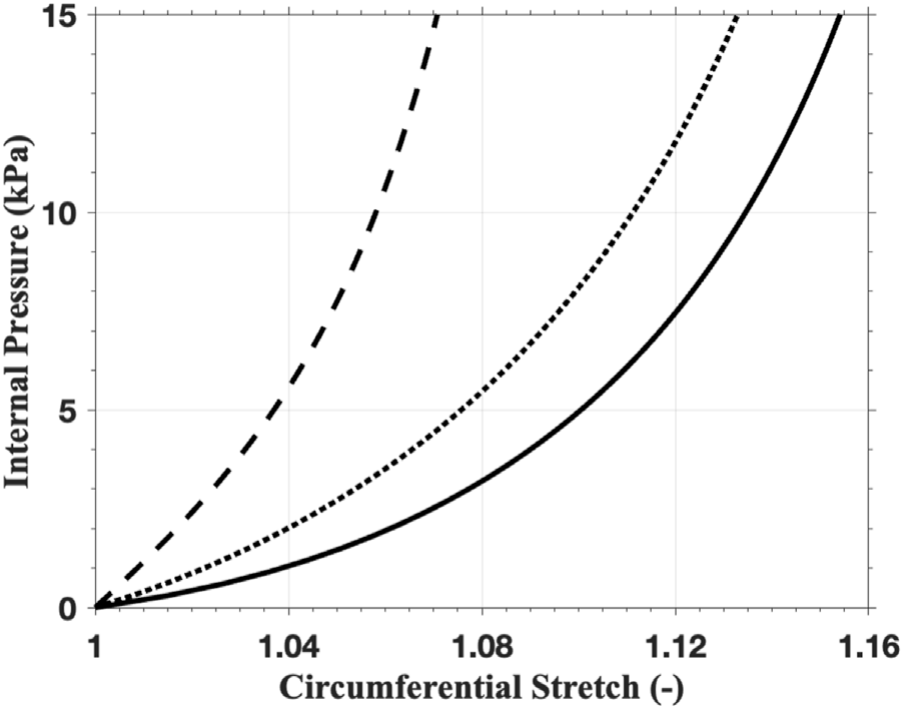
The mechanical response of the thin-walled tube with the anisotropic material model using the parameters of intima (solid line), media (dashed line), and adventitia (dotted line).

Solving (Equations 5, 6) with a series of prescribed values of $p^{\text{in}}$ , we obtain a plot of $p^{\text{in}}$ versus $\lambda_{\text{c}}$ , as shown in Figure 8. Among the three layers, the media exhibits the strongest mechanical response to the loads under the same circumferential stretch. This is primarily due to the fact that the mean orientation of the media is more aligned to the circumferential direction than in the other two layers, and the fibers are less dispersed in the media.

**FIGURE 9:**
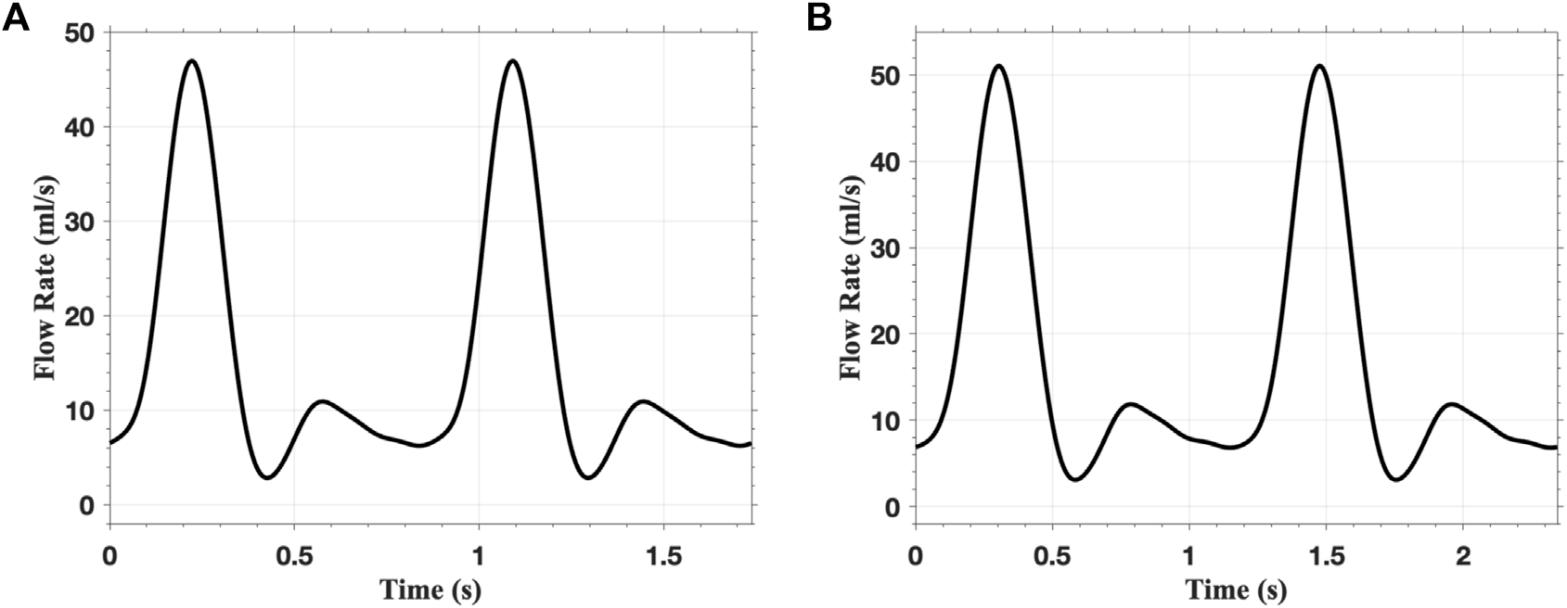
The flow rate applied at the inlet of **(A)** fAAA and **(B)** sAAA for two cardiac cycles.

**FIGURE 10:**
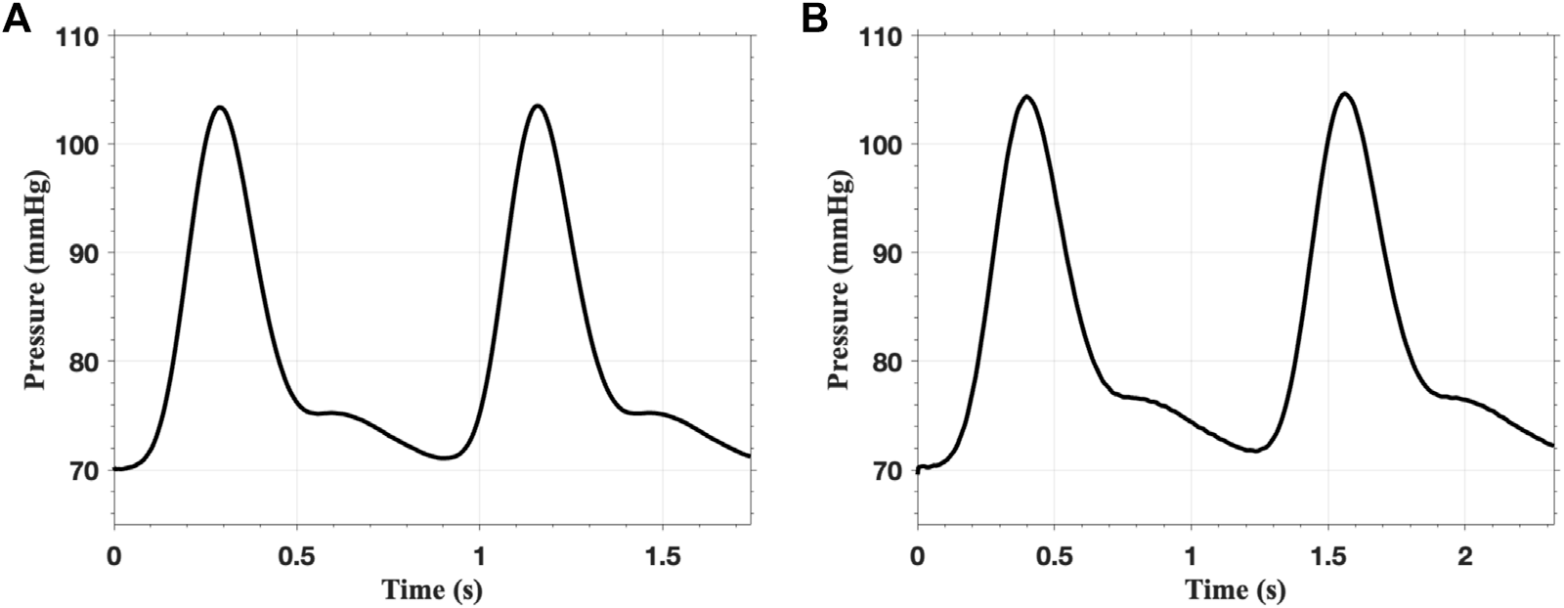
The pressure on the outlet of **(A)** single-layered fAAA and **(B)** single-layered sAAA for two cardiac cycles.

In the FSI analysis, the inlet flow boundary condition is depicted in Figure 9. The Vascular Model Repository provides the original inlet flow boundary condition at the proximal end of the descending aorta (VMR, 2024). The original flow rate is scaled based on the blood flow distribution ratio to determine the inlet flow rate of the AAAs. In this study, the distribution ratio is taken as 28.2% for both AAA models. The parameters for the outflow boundary condition are listed in Table 2. The parameters of the lumped parameter model are determined through parameter tuning, ensuring that the outlet pressure remains within the physiological range. The outlet pressure curves, shown in Figure 10, follow a similar pattern across all cases in this study, with the pressure value ranging from 70 mmHg (diastolic pressure) to 105 mmHg (systolic pressure), reflecting typical physiological conditions.

**TABLE 2:** The parameters for resistance boundary condition at the outlet.

	$R\left(g\cdot s^{-1}\cdot {cm}^{-4}\right)$	$P\left(g\cdot s^{-2}\cdot {cm}^{-1}\right)$
fAAA	1,662.96	82,468.59
sAAA	1,667.56	81,835.74

For the fAAA (sAAA) cases, the time step size is set to be $5.0\times 10^{-5}s$ $\left(6.8\times 10^{-5}s\right)$ . All displayed results are obtained at the moment when the tissue reaches its peak stress during the second cardiac cycle. Mesh independence has been confirmed, and we present the results from the finest mesh. The mesh details are listed in Table 3.

**TABLE 3:** The spatial discretization for different cases.

	Number of fluid elements	Number of solid elements
fAAA without ILT	$1.29\times 10^{6}$	$9.98\times 10^{5}$
fAAA with the original volume of ILT	$7.21\times 10^{5}$	$1.51\times 10^{6}$
fAAA with a smaller volume of ILT	$1.10\times 10^{6}$	$1.18\times 10^{6}$
sAAA without ILT	$1.32\times 10^{6}$	$9.58\times 10^{5}$
sAAA with the original volume of ILT	$1.15\times 10^{6}$	$1.36\times 10^{6}$
sAAA with a smaller volume of ILT	$1.38\times 10^{6}$	$1.15\times 10^{6}$

### 3.1 Multi-layered AAA tissue model

**FIGURE 11:**
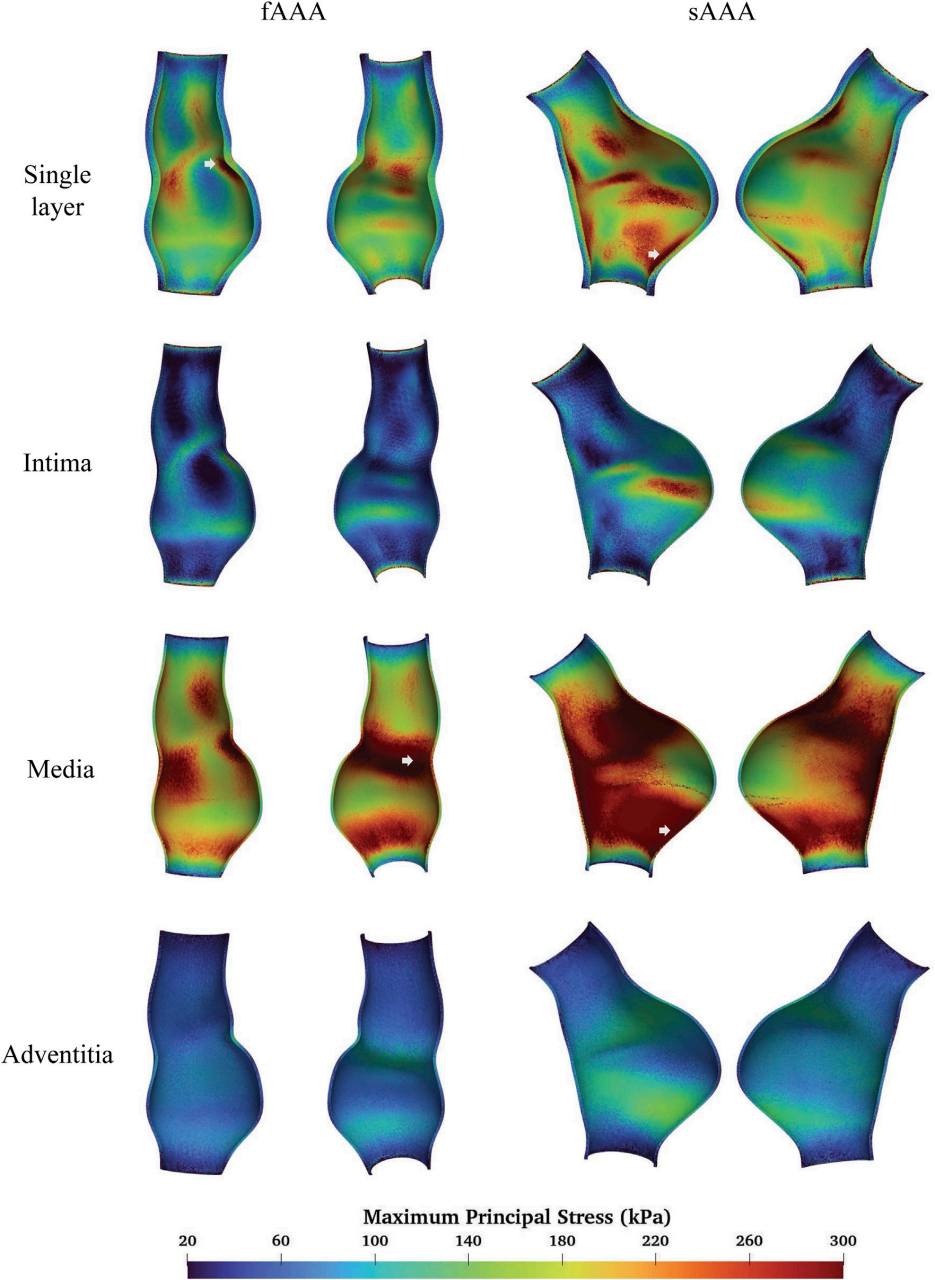
The MPS given by the single-layered and multi-layered tissue models and the location of maximum MPS.

We compare the FSI analysis results of AAAs with the vessel wall modeled as either multi-layered or single-layered tissue. The MPS distributions are shown in Figure 11. Note that both sides of the cross-sectional plane are illustrated in the visualization of the stress distribution in this and subsequent figures. The single-layered model exhibits a smooth transmural stress distribution, with the stress decreasing from the interior side to the exterior side of the tissue wall. In contrast, the multi-layered model indicates that the stress is significantly higher in the media compared to the rest two layers. For fAAA, the single-layered model predicts an average stress of 90.80kPa , while the multi-layered tissue model gives an average stress of 39.85kPa in the intima, 167.23kPa in the media, and 52.65kPa in the adventitia. For sAAA, the single-layered tissue model predicts an average stress of 116.22kPa , whereas the multi-layered tissue model gives average stresses of 62.47kPa in the intima, 207.54kPa in the media, and 69.45kPa in the adventitia. In the multi-layered model, the media bears the majority of the stress, with the stress distribution within the three layers exhibiting a similar pattern. Therefore, in this and subsequent sections, we focus on the stress distribution in the media, as it is representative of the stress distribution in the multi-layered tissue model.

When predicting the region of high stress, both single-layered and multi-layered models yield similar results, with high-stress areas primarily located near the proximal and distal ends of the aneurysms and lower stress observed within the aneurysms themselves. Figure 11 highlights the regions of the maximum MPS with arrows, showing that both models predict the location of these high-stress regions similarly. For fAAA (sAAA), the maximum MPS is 451.57kPa (525.85kPa) in the single-layered model and 698.91kPa (774.38kPa) in the multi-layered model. While both models deliver similar predictions in the region of high stress, the maximum MPS predicted by the multi-layered tissue model is notably higher compared to the single-layered model.

### 3.2 ILT

To study the impact of ILT on the aneurysm, we consider the following four cases:

fAAAw1: fusiform AAA with the original volume of ILT;
fAAAw2: fusiform AAA with a reduced volume of ILT;
sAAAw1: saccular AAA with the original volume of ILT;
sAAAw2: saccular AAA with a reduced volume of ILT.

**FIGURE 12:**
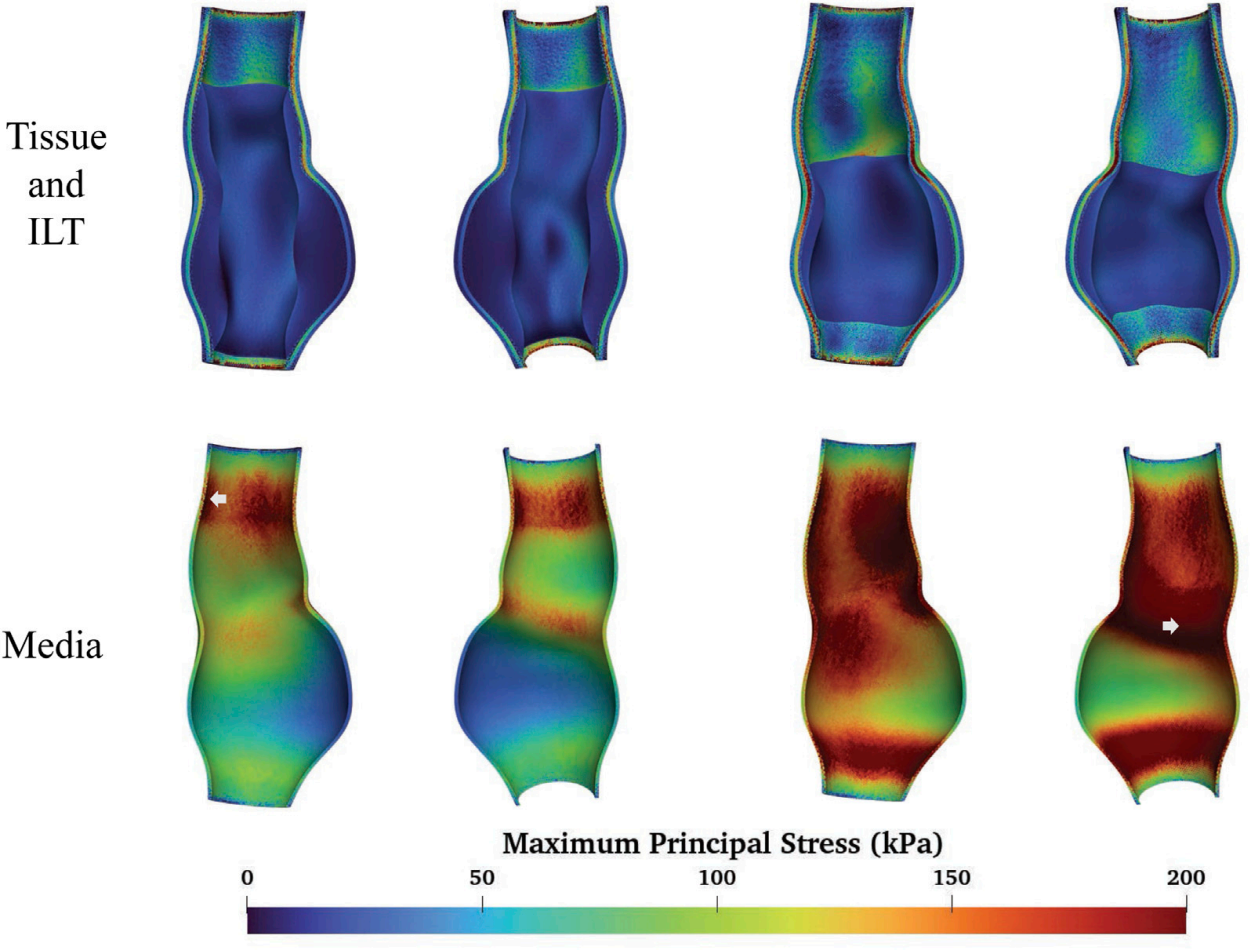
The MPS of fAAAs with ILT and the location of maximum MPS.

**FIGURE 13:**
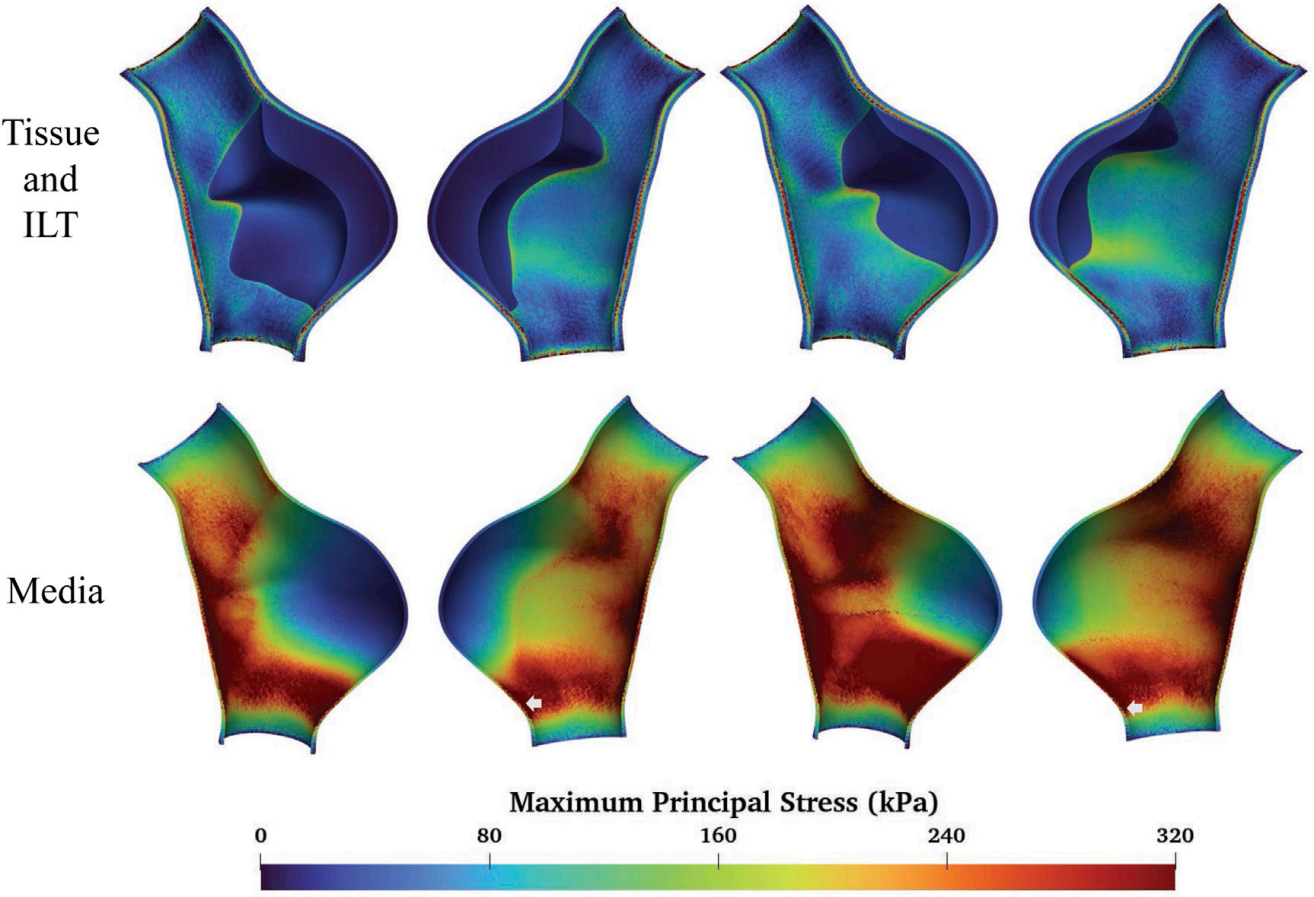
The MPS of sAAAs with ILT and the location of maximum MPS.

Figures 12, 13 depict the distribution of the MPS in both the ILT and aneurysm tissue. It can be observed that the MPS within the ILT is relatively low, decreasing smoothly from the luminal surface towards the abluminal surface. Table 4 presents the average MPS in the ILT and different tissue layers across the different cases. As shown in the table, the average stress level in the ILT is lower than that in the intima and adventitia. Again, the media exhibits the highest stress among the three layers.

**TABLE 4:** The average MPS of ILT, intima, media, and adventitia, with units in kPa .

	ILT	Intima	Media	Adventitia
fAAAw1	13.53	17.38	75.07	25.59
fAAAw2	17.79	30.50	134.55	43.31
sAAAw1	15.57	36.04	144.15	46.99
sAAAw2	22.75	50.40	185.16	61.10

**FIGURE 14:**
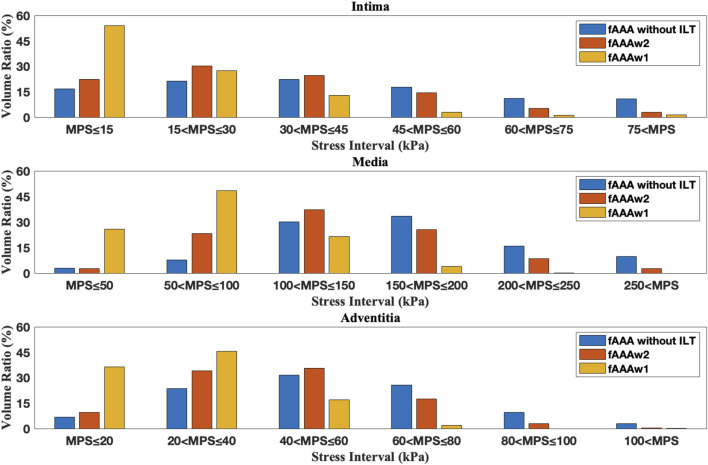
The volume ratio of stress intervals for multi-layered fAAA without ILT, fAAAw1, and fAAAw2.

**FIGURE 15:**
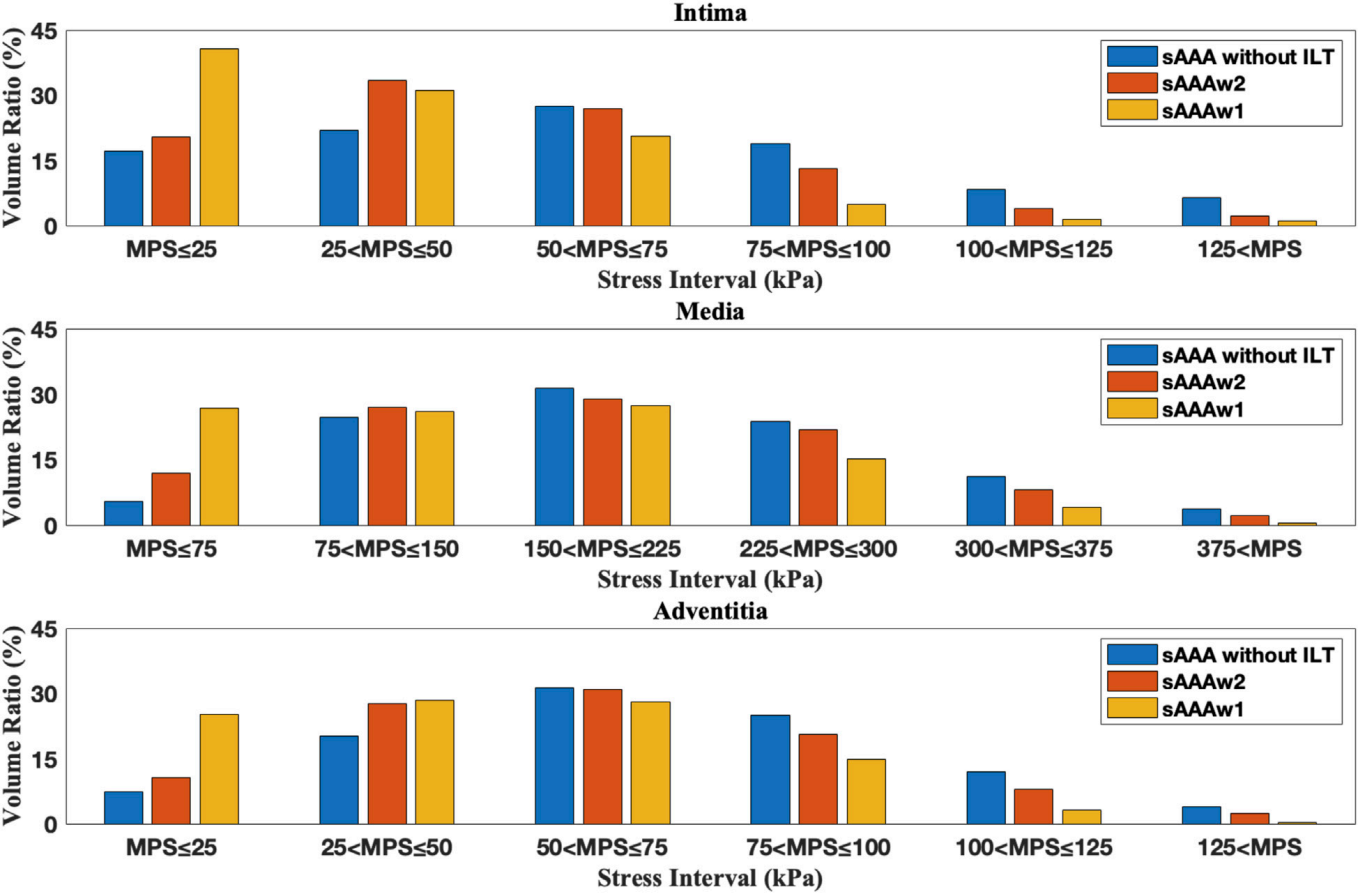
The volume ratio of stress intervals for multi-layered sAAA without ILT, sAAAw1, and sAAAw2.

As shown in Figures 12, 13, compared to the results of Section 3.1, the presence of ILT significantly reduces the stress within the aneurysm tissue, leading to decreases in average stress in the media by 55.08% for fAAAw1, 19.49% for fAAAw2, 30.54% for sAAAw1, and 10.78% for sAAAw2. This indicates that ILT effectively reduces the stress in the AAA tissue. The volume ratios across series of stress intervals are further analyzed. For a specific tissue layer, the volume ratio of a stress interval is defined by $V/V_{\text{total}}\times 100\%$ , where V represents the total volume of elements within a specific stress range, and $V_{\text{total}}$ denotes the total volume of all elements. As shown in Figures 14, 15, ILT significantly reduces the volume ratio of high-stress elements and increases the volume ratio of low-stress elements in all three tissue layers. Moreover, the ILT with its original volume (fAAAw1 and sAAAw1) exhibits a more pronounced effect compared to the ILT with a smaller volume (fAAAw2 and sAAAw2). Arrows in Figures 12, 13 highlight the region of the peak stress. For the two fAAA cases, the stress distribution is notably affected by the presence of ILT, with a significant shift in the region of the maximum MPS. The maximum MPS values are 292.49kPa for fAAAw1 and 527.69kPa for fAAAw2. In the sAAA cases, the ILT does not significantly impact the overall stress distribution within the aneurysm, and the site of the peak stress remains unchanged. The maximum MPS values are 547.62kPa for sAAAw1 and 654.80kPa for sAAAw2, which are comparable to the peak stress value obtained from the sAAA with multi-layered tissue model in Section 3.1.

### 3.3 Media degradation

**FIGURE 16:**
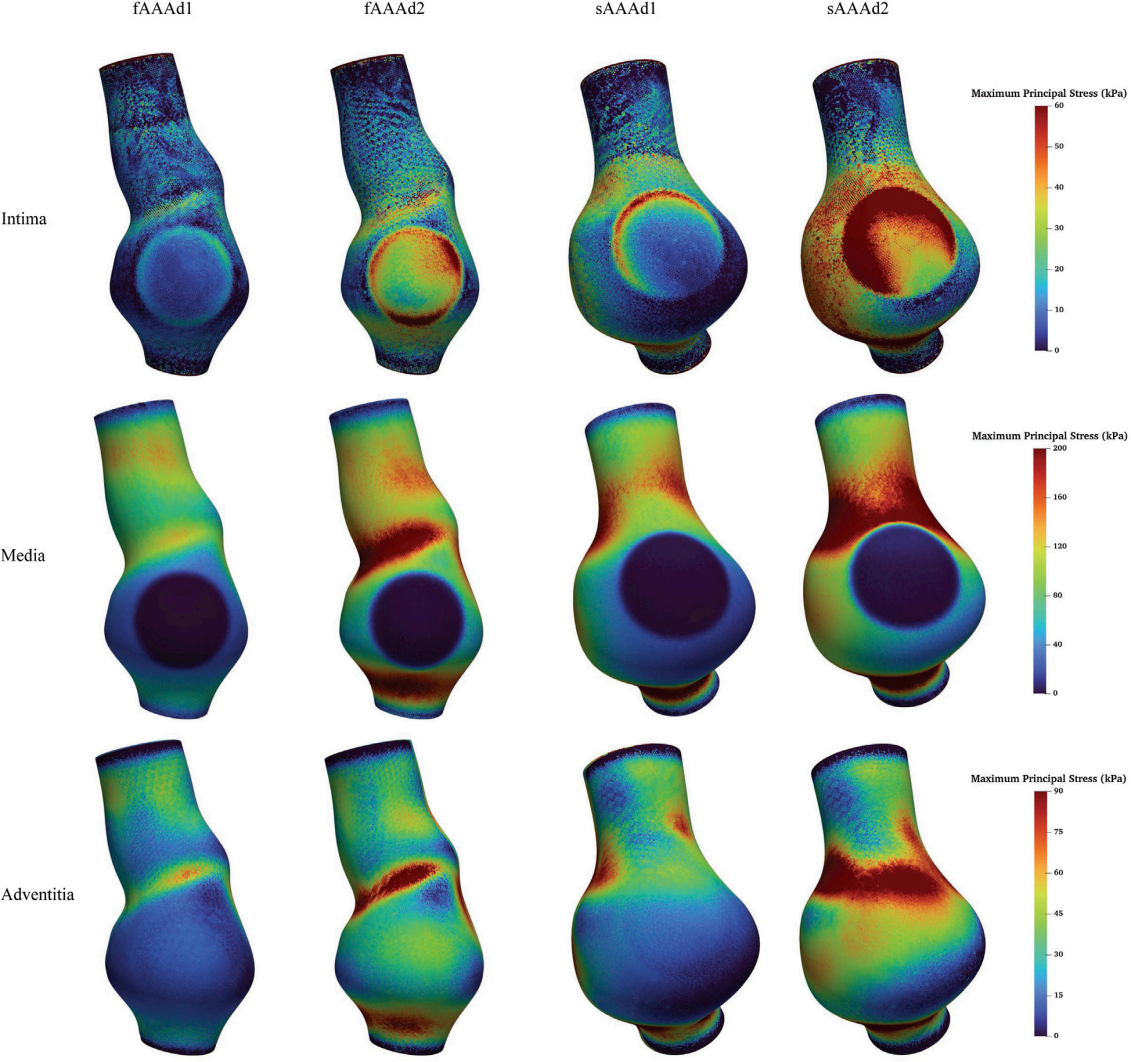
The MPS in the cases with media degradation.

**FIGURE 17:**
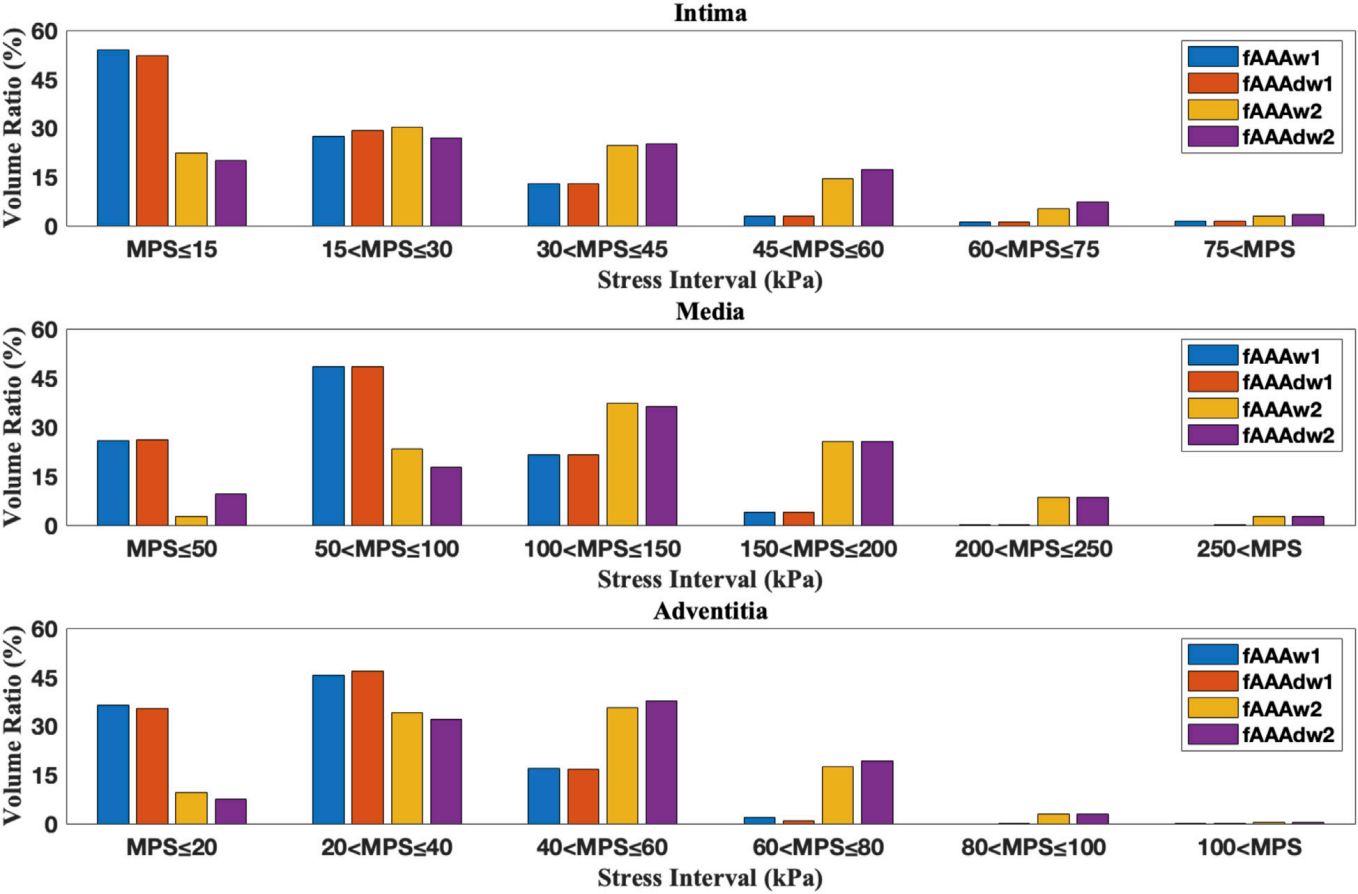
The volume ratio of stress intervals for fAAAw1, fAAAw2, fAAAdw1, and fAAAdw2.

**FIGURE 18:**
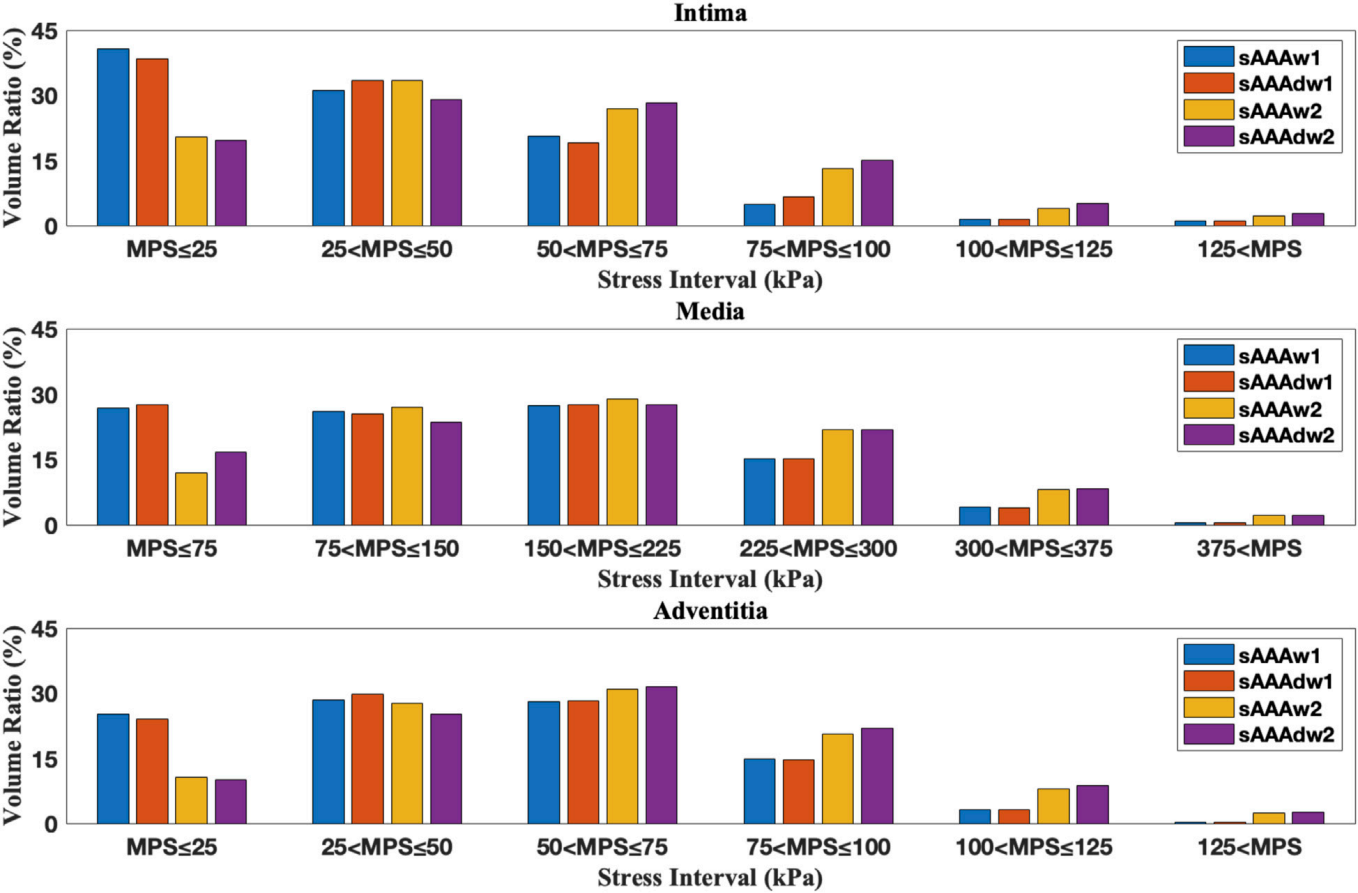
The volume ratio of stress intervals for sAAAw1, sAAAw2, sAAAdw1, and sAAAdw2.

We further examine the degradation of the media induced by ILT. In regions with thick ILT, degradation zones are virtually designated in the media. The examples in this section, named fAAAd1, fAAAd2, sAAAd1, and sAAAd2, correspond to the cases fAAAw1, fAAAw2, sAAAw1, and sAAAw2 from Section 3.2, with the introduction of degradation zones. As shown in Figure 16, the stress within the degradation zones of the media is significantly lower than that in the surrounding areas. In contrast, for the intima and adventitia, the stress within the degradation zones is slightly higher than in the neighboring areas. Table 5 presents the average MPS values for each tissue layer before and after introducing the degradation zones, along with the respective differences. The results indicate that the stress in the media significantly decreases in the degradation zones, while the stress in the rest two layers increase. Additionally, Table 6 presents the maximum MPS values for intima and adventitia before and after introducing the degradation zones, along with the respective differences. Tables 5, 6 show that, in cases with larger ILT volumes, the stress increase in the intima and adventitia is smaller than in cases with smaller ILT volumes. This suggests that, under similar degradation conditions, a thicker ILT mitigates the weakening effect on the tissue caused by degradation. Additionally, we further investigate the influence of the degradation zone on the volume ratios. Figures 17, 18 show that media degradation has minimal effect on the volume ratios across all three tissue layers. Consequently, the impact of the degradation zone is localized.

**TABLE 5:** The average MPS of intima, media, and adventitia in the media degradation cases, with units in kPa .

	fAAAw1	fAAAd1	Difference
Intima	6.02	11.19	5.17
Media	28.38	4.22	−24.16
Adventitia	10.41	15.37	4.96

	fAAAw2	fAAAd2	Difference
Intima	21.89	47.13	25.24
Media	79.93	14.95	−64.98
Adventitia	27.45	47.93	20.48

	sAAAw1	sAAAd1	Difference
Intima	11.66	19.49	7.83
Media	40.94	6.38	−34.56
Adventitia	17.26	24.72	7.46

	sAAAw2	sAAAd2	Difference
Intima	39.68	74.01	34.33
Media	107.31	19.56	−87.75
Adventitia	45.01	74.28	29.27

**TABLE 6:** The maximum MPS of intima and adventitia in the media degradation cases, with units in kPa .

	fAAAw1	fAAAd1	Difference
Intima	23.88	37.80	13.92
Adventitia	23.16	34.21	11.05

	fAAAw2	fAAAd2	Difference
Intima	74.64	116.61	41.97
Adventitia	65.74	113.62	47.88

	sAAAw1	sAAAd1	Difference
Intima	38.89	79.82	40.93
Adventitia	52.03	64.31	12.28

	sAAAw2	sAAAd2	Difference
Intima	119.87	191.06	71.19
Adventitia	128.30	205.99	77.69

## 4 Discussion

Previous studies have applied layered models for stress analysis in cardiovascular diseases, and they reported similar stress distributions across the tissue layers, with the media experiencing the highest stress, followed by the adventitia, and then the intima (Gao et al., 2013; Simsek and Kwon, 2015; Fan et al., 2024). This suggests that the media serves as the primary load-bearing layer of the vessel under physiological loading. Likewise, the work of Simsek suggests that when the media degrades, the intima and adventitia take on a greater load (Simsek and Kwon, 2015), aligning well with our findings. According to Sassani, in the case of three-layered wall rupture, the stress in the adventitia exceeds that in the media, with both layers bearing higher stress than the intima. This highlights the adventitia’s role in maintaining vascular structural integrity when the media degrades (Sassani et al., 2015). From this discussion, it is clear that compared to single-layered tissue models, multi-layered tissue models provide valuable insights into the biomechanical responses of the individual layers and their distinct roles in bearing physiological loads. Therefore, when analyzing stress distributions in vascular tissues, a physiologically detailed model should be considered. However, we also note that there is no significant difference in the time-averaged wall shear stress between the single-layered and multi-layered models, indicating that hemodynamic forces are less sensitive to the choice of the tissue model. This is consistent with our prior study (Sun et al., 2025a).

Numerous studies have investigated the biomechanical effects of ILT, especially regarding its impact on the location of the maximum stress. Throop et al. emphasized that it can significantly alter the location of the peak stress (Throop et al., 2022). Riveros et al. found that the region of the maximum stress often coincides with the thinnest part of the ILT (Riveros et al., 2015). Conversely, Xenos et al. reported that ILT had little impact on the location of the maximum stress (Xenos et al., 2015). Our findings suggest that whether ILT affects the location of the maximum MPS depends on whether the ILT sufficiently covers the area of the peak stress. These varying results underscore the complex role ILT plays in aneurysm biomechanics and highlight the need for further patient-specific studies to better understand its effects on stress distribution and rupture risk.

Our study demonstrates that, under equivalent conditions of degradation, a thinner ILT provides significantly less biomechanical protection to the affected region compared to a thicker ILT. However, in the specific cases analyzed in this study, even with increased stress levels observed in the intima and adventitia after degradation, the stress levels remained lower compared to the failure stress reported by Sassani et al. (2015), which are 276.8 kPa (axial) and 511.1 kPa (circumferential) for intima, 957.5 kPa (axial) and 1728.2 kPa (circumferential) for adventitia, even when the ILT was relatively thin. This suggests that, in these scenarios, the presence of a thinner ILT does not substantially increase the rupture risk. Since our model constructed the degradation regions virtually, we believe that more sophisticated models are needed to explore the impact ILT might have on aneurysm rupture. This should include accounting for the local thickness of the ILT (Vorp et al., 2001) and the age of ILT formation (Tong et al., 2011). These factors are crucial for accurately predicting the protective or detrimental effects of ILT on aneurysm stability.

## 5 Conclusion

In this study, we investigated the biomechanical behavior of AAA by incorporating the layered architecture of the vascular wall, anisotropic material properties, and the effects of ILT, particularly its role in media degradation. Through detailed FSI analysis of both fusiform and saccular AAAs, we compared the MPS distribution under various conditions: with ILT, with ILT but no degradation, and with both ILT and degradation. The results offer valuable insights into the stress variations within each layer of the aneurysm wall, enhancing our understanding of AAA biomechanics and the potential impact of ILT on aneurysm progression.

The multi-layered AAA tissue model, compared to the single-layer model, offers a more detailed transmural stress distribution, with the media serving as the primary load-bearing component of the aneurysm tissue. Moreover, the presence of ILT significantly reduces the stress levels in the aneurysm wall beneath it. However, ILT does not necessarily affect the location of the maximum stress. Degradation of the media increases stress levels in both the intima and adventitia.

In the future, we will build upon the current multi-layered anisotropic hyperelastic model by incorporating a viscoelastic model to better capture the biomechanical properties of vascular tissues. By combining this approach with existing FSI tools, we will be able to more accurately model cardiovascular and cerebrovascular diseases, thereby enhancing our understanding of the biomechanical mechanisms underlying these conditions.

## 6 Limitations

The sample size used in the experiments is relatively small. While the data collected provide valuable insights into the studied topic, the limited sample size may affect the generalizability of the findings to a broader population. Specifically, the small sample size introduces a risk of bias due to individual differences. For instance, AAAs with a maximum diameter exceeding 5.5 mm were not included in this study, which may limit the applicability of our results to small AAAs. Nevertheless, the main conclusions of this study remain consistent with findings from existing studies (Simsek and Kwon, 2015; Xenos et al., 2015; Riveros et al., 2015).

Furthermore, for the purpose of using FSI to diagnose AAA, obtaining additional patient-specific information, such as material properties, is essential and challenging. In this study, we used the same material parameters derived from experimental data to describe ILT and AAA tissue (Di Martino and Vorp, 2003; Sassani et al., 2015). However, as the results obtained using the multi-layered model align with both the functional roles of different layers (Holzapfel et al., 2000) and the experimental findings (Sassani et al., 2015), the conclusions of this study remain reliable.

## Data Availability

The raw data supporting the conclusions of this article will be made available by the authors, without undue reservation.

## Footnotes

1 The names of the fAAA and sAAA models are 0040_H_ABAO_AAA and 0042_H_ABAO_AAA, respectively, in the Vascular Model Repository.
